# Restless Legs Syndrome Among Sudanese Patients With Type 2 Diabetes Mellitus: A Case-Control Study

**DOI:** 10.7759/cureus.9635

**Published:** 2020-08-09

**Authors:** Hyder Mirghani

**Affiliations:** 1 Internal Medicine, Tabuk University, Tabuk, SAU

**Keywords:** type 2 diabetes, glycated hemoglobin, complications, sudan, restless leg syndrome

## Abstract

Background

There is increasing awareness about the association of restless legs syndrome (RLS) with type 2 diabetes. This study assessed RLS and its associations among patients with diabetes.

Material and methods

This case-control study was conducted among 160 subjects (82 patients with diabetes and 78 controls) attending a diabetic clinic in Omdurman, Sudan, during the period from June 2018 to September 2019. A structured questionnaire was used to collect demographic factors, diabetic neuropathy, nephropathy, retinopathy, and macrovascular complications. The neck circumference was measured to assess adiposity, and a blood sample was taken for the glycated hemoglobin (HbA1_c_) estimation. The local ethical committee approved the research, and the Statistical Package for Social Sciences (SPSS; IBM Corp., Armonk, NY) was used for data analysis. A P-value of <0.05 was considered significant.

Results

There were 82 patients with type 2 diabetes and 78 controls matched for age and sex. Restless legs syndrome was higher among patients with diabetes (31.7% vs. 10.3%%) with a significant statistical difference, P-<0.05. A direct positive relationship was found between restless legs syndrome and diabetic neuropathy (Wald=5.48, P-value=0.019, 95%CI 1.70-410.76), no relationship was found between RLS, diabetic retinopathy, glycated hemoglobin, sex, and neck circumference, P-values (0.757, 0.804, 0.317, and 0.361 respectively).

Conclusion

Restless legs syndrome was prevalent among patients with type 2 diabetes and was more common among patients with diabetic neuropathy, no relationship was found between restless legs syndrome, age, sex, neck circumference, HbA1_c_, and retinopathy

## Introduction

Restless legs syndrome (RLS) is one of the most common neurologic disorders, affecting up to 8% of adults in developed countries, and clinically significant restless legs syndrome is estimated to affect 2%-3% of the adult population [1-2]

Restless legs syndrome is a relatively common morbid and treatable disorder although it is commonly overlooked due to it's a typical presentation [3].

The pathophysiology is complex, involving an iron deficiency in the dopamine neurons of the substantia nigra. These neurons project to the striatum, which plays a crucial role in movement modulation. The iron levels are usually normal outside the brain. Several genes (BTBD9, Btbd9, and MEIS1) were identified as increasing the risk of restless legs syndrome in animal models [4]. Neuroimaging of the brain (magnetic resonance imaging (MRI), positron emission tomography (PET), and single positron emission computed tomography (SPECT) also demonstrated the low iron not only in the substantia nigra but extending to involve the thalamus. The low iron concentration leads to nigrostriatal and mesolimbic dopaminergic pathways and, in turn, to sensorimotor and limbic nociceptive pathways [5].

Recent literature showed the involvement of other areas not subserving movement or sensation (cingulate cortex and cerebellum). The pre and postcentral gyri and cerebellum were also affected and activated. In addition to the dopaminergic oxygen-sensing system, the opioid, glutamatergic, and serotonergic systems are involved [6].

Clinically, restless legs syndrome is characterized by an urge to move the limbs accompanied by an unpleasant sensation that increases at night and is relieved by movement. RLS could be primary or secondary to various disorders, including iron deficiency anemia and polyneuropathy. RLS and painful diabetic neuropathy, although similar, are different entities that pose a great diagnostic challenge [7-8]. The diagnosis is primarily clinical, and there are no diagnostic tests. The executive committee of the International Restless Legs Syndrome Study and Workshop group developed and approved four criteria that must be all present more than five times/month [9].

Diabetes mellitus is a global health burden. Currently, 285 million are affected by the disease, and the projection for the year 2030 is 438 million, with Asian countries suffering the bulk of the total diabetes epidemic [10].

Most patients are diagnosed with diabetes mellitus after the age of 40 years, but despite the improvement in diabetes management, comorbidities are high from its microvascular complications, mainly neuropathy and nephropathy, and most die from cardiovascular disease [11-12].

The American Diabetes Association's recommendation is to target glycosylated hemoglobin (HbA1_c_) to less than seven to reduce microvascular complications in type 1 and type 2 diabetes. If metformin is used in overweight diabetic patients, the risk of myocardial infarction is also reduced [13-14].

Previous studies observed that patients with diabetes have 4-4.4 times more risk of developing restless legs syndrome than the general population although others observed no relationship between the syndrome and diabetes mellitus. A significant association between this morbid disorder and diabetes is not unusual because of the etiologic role of diabetes in producing renal failure and polyneuropathy [9,15-16]. Diabetes mellitus and restless legs syndrome when observed together could precipitate the other deleterious effects. Because of the controversy about the association of restless legs syndrome and diabetes and the paucity of literature regarding this relatively common syndrome, studies conducted in other countries may not apply to Sudan. Thus, we conducted this search. In the current study, we assess the restless legs syndrome among patients with type 2 diabetes mellitus.

## Materials and methods

This case-control study conducted a diabetes clinic at Omdurman Teaching Hospital, Sudan during the period from June 2018 to September 2019. Eighty-two patients with type 2 diabetes diagnosed according to American Diabetes Association Guidelines 13 and 78 healthy controls who were recruited from the co-patients (to minimize socio-economic differences) were approached. Patients with other sleep disorders, renal failure, anemia, or severe hypoglycemia were excluded. A structured questionnaire was used to interview the study sample to collect the following information. Age, sex, diabetes, and microvascular complications, including neuropathy, nephropathy, and retinopathy, macrovascular complications, diabetic complications, including nephropathy, were collected from the patient's records. Microalbuminuria was adopted for the diagnosis of diabetic nephropathy. The Restless Syndrome International Study Group Guidelines were used to diagnose restless leg syndrome [8-9]. The criteria include the presence of an unpleasant sensation in the limb with a desire to move, the unpleasant sensation increases with rest and at night and decreases with movement. The four components should be positive for the diagnosis. The diagnosis of peripheral neuropathy was based on history and abnormal clinical tests, including touch and pain sensation, position and vibration sense, and ankle reflex. Nerve biopsy was not done to diagnose small fiber neuropathy. Neck circumference was measured below the laryngeal prominence and perpendicular to the long axis of the neck, and the minimal circumference is recorded to the nearest 0.1 cm. The patients should look forward and the shoulders not elevated. Previous literature concluded that neck circumference >37 in males and 34 cm in females are probably the best cut-offs to determine patients with central adiposity [17-18]. A blood sample was taken for HbA1_c_ measurement to assess the degree of diabetes control using the reagent from using a glycol hemoglobin reagent set from HB1C Siemens Healthcare Diagnostics Newark, DE 19714, USA.

The ethical committee of Elnour Complex, Omdurman, Sudan, approved the research.

The Statistical Package for Social Sciences (SPSS, version 20, IBM Corp, Armonk, NY) was used for data analysis. The chi-square (for categorical data), independent sample t-test (for numerical values), and logistic regression analysis were used for data analysis. The data were presented as percentages and mean±SD, a P-value of <0.05 was considered significant.

## Results

They were 160 subjects, 82 patients with type 2 diabetes and 78 control subjects matched for age and sex (63.4% vs. 51.3%) were females with no significant statistical difference, their ages were (54.02±7.55) years for patients with diabetes and (50.94±11.30) for control subjects with no significant statistical difference, P-value=0.155, the restless legs syndrome was higher among patients with diabetes (31.7% vs. 10.3%%) with a significant statistical difference, P-value=0.019. Table 1 depicted a comparison between patients with diabetes and control subjects.

**Table 1:** A comparison between patients with type 2 diabetes and control subjects

Character	Diabetes	controls	P-value	95%CI
Age	54.02±7.55	50.94±11.30	0.155	-1.18-7.33
Restless legs score	1.49±1.81	0.45±1.10	0.003	0.37-1.71
Sex: Males; Females	30 (36.6%); 52 (63.4%)	38 (48.7%); 40 (51.3%)	0.273	0.249-1.48
Restless legs syndrome	26 (31.7%)	8 (10.3%)	0.019	1.19-13.84
Abnormal neck circumference	60 (81.1%)	42 (26.9%)	0.012	1.30-10.35

Table 2 showed a comparison between patients with diabetes mellitus and restless legs syndrome and their counterparts without RLS. RLS was commoner among patients with peripheral neuropathy (69.2% vs. 14.8) with a significant statistical difference, P-value=001, no significant statistical difference was evident regarding diabetic retinopathy, age, sex, and abnormal neck circumference. However, a higher numerical value was observed regarding the glycated hemoglobin (10.04±2.53 vs. 8.51±1.62, P-value=0.077).

**Table 2:** The relationship of the restless legs syndrome (No=26) with diabetes neuropathy, retinopathy, and sex among the study group

Character	RLS=26	No RLS=54	P-value	95%CI
Age	53.92±9.33	54.00±6.89	0.977	-5.37-5.21
Sex: Males; Females	6 (23.1.8%); 20 (76.9%)	24 (44.4%); 30 (55.5%)	0.298	0.883-2.17
Neuropathy	18 (69.2%)	8 (14.8%)	0.001	0.16-0.377
Retinopathy	6 (23.1%)	6 (11.5%)	0.321	0.072-2.42
Abnormal neck circumference	24 (92.3%)	36 (75%)	0.199	0.42-37.55
HbA1_c_	10.04±2.53	8.51±1.62	0.077	-.175-3.22

A direct positive relationship was found between restless legs syndrome and diabetic neuropathy (Wald =5.48, P-value=0.019, 95%CI 1.70-410.76), no relationship was found between RLS, diabetic retinopathy, HbA1_c_, sex, and neck circumference, P-values (0.757, 0.804, 0.317, and 0.361, respectively). See Table 3.

**Table 3:** The relationship of the restless legs syndrome (No=26) with sex, neck circumference, diabetes neuropathy, retinopathy, and HbA1c among the study group HbA1_c_: glycated hemoglobin

Character	Wald	df	P-value	95%CI
Neuropathy	5.48	1	0.019	1.70-410.76
Retinopathy	0.096	1	0.757	0.118-18.84
HbA1_c_	0.061	1	0.804	0.533-1.62
Sex	1.000	1	0.317	0.46-10.95
Neck circumference	0.834	1	0361.	0.033-3.61
Constant	0.005	1	0.946	

## Discussion

Several studies have documented the high prevalence of restless legs syndrome among patients with type 2 diabetes; the association cannot be explained by the presence of peripheral neuropathy alone. Another theory is the prolonged sleep loss that could lead to insulin resistance and metabolic disturbances [19]. In the present study, restless legs syndrome was found in 31.7% of patients as compared to 10.3% of control subjects. The present finding was higher than the result of Harashima et al. (8%) [20]. The current findings were similar to a study published by Zobeiri et al. who concluded a prevalence of 28.6% as compared to 7.1% in control subjects [21]. Previous studies from other countries found lower rates of RLS among patients with diabetes [22-23]. A previous study showed no association of restless legs syndrome with type 2 diabetes in contradiction to the current findings [24]. The different rates of RLS may be explained by other comorbidities and study types [25]. In the current study, peripheral neuropathy was associated with restless legs syndrome in line with a previous observation [20]. The symptoms of restless legs syndrome and peripheral neuropathy are similar and aggravate each other deleterious effects; this similarity may lead to misdiagnosis and delayed treatment. Furthermore, small fiber neuropathy may be a risk factor for restless legs syndrome [26-28]. The current study findings imply the importance of the earlier detection of restless legs syndrome because it is a treatable condition that aggravates diabetic neuropathy and negatively affects the quality of life and sleep. Previous researchers concluded that restless legs syndrome through sleep disturbances and psychological factors could impair glycemic control [29], however, the current finding of a numerically higher (not reaching statistical difference) glycated hemoglobin among patients with restless legs syndrome contradict these observations. In the present study, no relationship was found between the restless legs syndrome, sex, retinopathy, and neck circumference in similarity to a survey carried out in Colombia [30] and concluded similar results. The present findings are in contradiction to Ruiz et al. who concluded a high neck circumference among restless legs syndrome patients [30]. Racial, behavioral, and environmental factors may explain the differences [30].

The study limitation is the fact that the survey was conducted at a single diabetes clinic so generalization cannot be insured. Further larger studies to investigate the risks associated with this common sleeping disorder are highly recommended. Raising the physician's awareness about this underdiagnosed syndrome could improve the management of patients with diabetes mellitus.

## Conclusions

Restless legs syndrome is prevalent in patients with type 2 diabetes and associated with peripheral neuropathy, which may pose great difficulty in the differentiation of these two common morbid disorders. Screening for this prevalent sleep disorder may need to be included in the diabetes holistic care. The differentiation of RLS from peripheral neuropathy is needed because the management is different.
